# Magnetic resonance imaging evaluation of the correlation between calcific tendinitis and rotator cuff injury

**DOI:** 10.1186/s12880-022-00746-0

**Published:** 2022-02-08

**Authors:** Xiao-Kun Yu, Jian Li, Le Zhang, Lei Li, Jin-Xing Li, Wen-Bin Guo

**Affiliations:** 1 Department of Radiology, The Fifth Centre Hospital of Tianjin, No. 41 of Zhejiang Road, Binhai New District, Tianjin, 300450 China; 2 Department of Radiology, Tianjin Hospital, Tianjin, 300211 China

**Keywords:** Shoulder joint, Calcific tendinitis, Rotator cuff tear, MRI, Hydroxyapatite

## Abstract

**Background:**

This study aims to evaluate the incidence of calcific tendinitis (CaT) in rotator cuff tears (RCTs) and to assess the correlation between CaT and RCTs with magnetic resonance imaging (MRI).

**Methods:**

The MRI of 108 patients with rotator cuff CaT admitted to our hospital from January 2019 to January 2021 were retrospectively analyzed. Another retrospective analysis was made of 108 patients with similar age, gender, occupation, and shoulder injury side to those in the first group. The incidence of RCTs and their correlation with CaT were assessed based on an MRI of shoulder joints.

**Results:**

There was a statistical difference (*p* < 0.05) in the incidence of RCTs between the CaT group (23.4%) and the control group (37.2%). No significant difference was observed in the size of the RCTs between the two groups (*P* = 0.422). In the CaT group, 17.4% of patients had complete tears, compared with 26.3% in the control group. There was no significant correlation between the calcification site and RCTs in the CaT group, and only 3.7% of patients suffered calcification and a tear in the exact location of the same tendon (*P* > 0.05, r = 0.03).

**Conclusions:**

Compared with patients with shoulder pain without CaT, patients with rotator cuff CaT suffered no increased risk of RCTs on MRI, so CaT and RCTs may have different pathological causes, and there is no significant correlation between the two.

## Background

Rotator cuff calcific tendinitis (CaT) is a disease characterized by calcium deposits in the rotator cuff tendon. It can affect any tendon in the rotator cuff, but the supraspinatus tendon is most commonly affected. Middle-aged female patients are susceptible to this disease, and the incidence in women is higher than that of men [1]. It has been previously reported that the incidence of CaT in asymptomatic individuals is 3%, and the incidence in patients with shoulder pain is 7% [2]. In magnetic resonance imaging (MRI) examinations of symptomatic or asymptomatic shoulder joints, the incidence of RCTs varies between 30 and 50% [3].

There is always controversy about the correlation between rotator cuff CaT and rotator cuff tears (RCTs). Calcific tendonitis and RCTs may provide a favorable condition for the development of each other and play a role in each other’s etiological mechanism [4]. Rotator cuff CaT can be accompanied by severe shoulder joint pain, resulting in a limited range of motion of the shoulder joint. Its clinical symptoms are similar to those of RCTs, which makes distinguishing between the two diseases a clinical challenge. Some scholars have found that about 25–28% of patients with CaT have RCTs in arthrography [5], whereas others have found that about 23% of patients with RCTs have calcification in their tendons [6, 7]. However, according to Verstraelen, Clavert, and Becciolini et al.‘s surgery-related research reports, RCTs rarely occur in CaT [8–10]. In the study of Becciolini et al., using ultrasound to evaluate patients with CaT of the rotator cuff, no cases of RCTs related to the calcified area were found [10]. We suspect that the results of previous studies with a correlation between CaT and RCTs are biased because these studies are based on surgical results, and only patients who fail to recover with conservative treatment will undergo surgical intervention. Therefore, it is unreliable to explain the relationship between CaT and RCTs based on the results of these studies [11]. For these reasons, further research is needed to clarify the probability of CaT and RCTs occurring at the same time and whether there is a causal relationship between the two.

A shoulder MRI is a good choice for this kind of evaluation and analysis because it can provide more accurate results [12]. Previously, there was limited literature discussing the relationship between CaT and RCTs with MRI. The purpose of this study is to investigate the incidence of RCTs in patients with CaT during shoulder MRI and the correlation between CaT and RCTs.

## Methods

### Research subjects

**Fig. 1:**
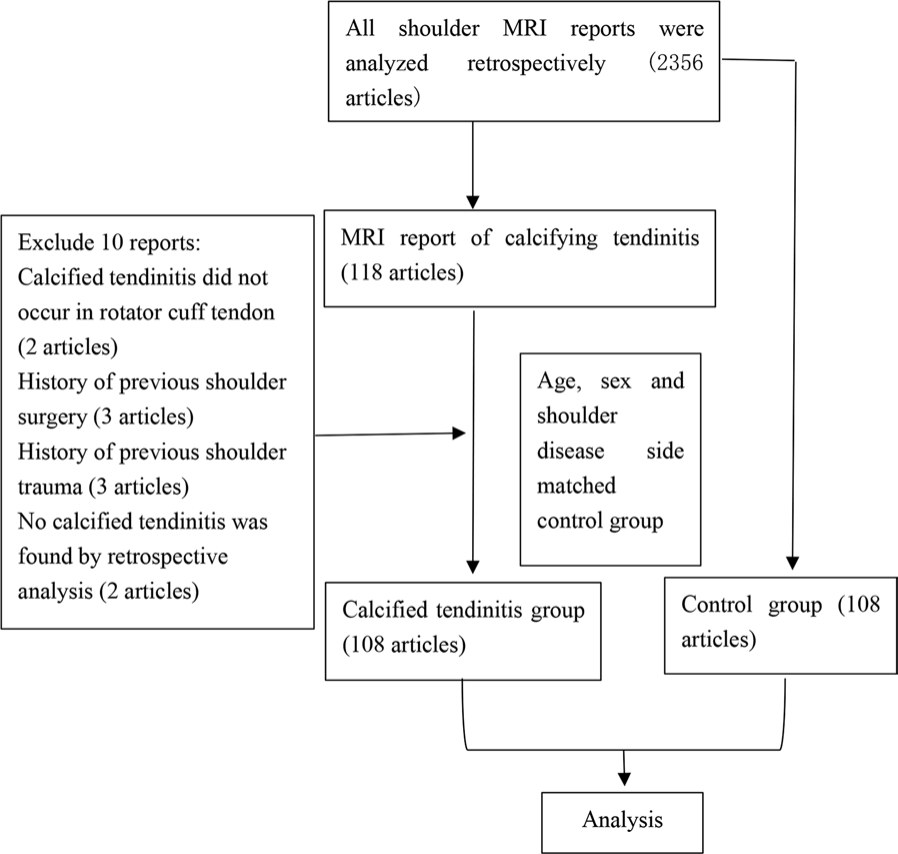
Screening flow chart of study subjects

This was a retrospective analysis of all MRI reports performed in our hospital from January 2019 to January 2021 due to shoulder pain. There were 2356 reports in total, of which 118 (5.0%) mentioned CaT. The inclusion criteria for CaT were: (1) MRI imaging showed that the calcified area of the tendon showed nodular or strip low signal in all sequences with clear and sharp boundaries; (2) The corresponding shoulder joint was confirmed by X-ray or CT as CaT; (3) When the tendon calcification range is large, generally less than 2 times the thickness of the tendon at the location. Among them, patients whose CaT was not in the rotator cuff tendon and patients with a history of shoulder surgery or shoulder joint trauma were excluded. In addition, there were two reports in which no CaT was found during retrospective analysis. According to these criteria, there were 108 shoulder MRI reports in total, and 108 patients were included in the CaT group. The control group consisted of 108 patients whose age, sex, occupations, and side of shoulder lesions matched those of the CaT group and whose MRI reports showed no CaT (Fig. 1). This study followed the Declaration of Helsinki and was approved by the Ethics Committee of the Fifth Centre Hospital of Tianjin. Written informed consent was obtained from all participants.

### Research methods

The shoulder sleeve tendons of each case of CaT lesions were evaluated against tearing as an indicator, and the size of the tendon tear (partial or complete tear) and its relationship with the calcification site (different tendons, different locations of the same tendon, the same tendon location) were recorded.

The rotator cuff tendon of each patient with CaT was evaluated with a tear as an indicator, and the size of the tendon tear (partial or complete tear) and the calcification site (different tendons, different locations of the same tendon, and the exact location of the same tendon) were recorded. Complete RCTs are RCTs that extends from the surface of the bursa to the articular surface. A partial tear refers to the RCTs that is only found on the surface of the bursa, the surface of the joint, or in the tendon. For cases of multiple CaT, the tendon with the largest calcification focus was chosen as the research object. In addition, each patient’s age, gender, and side of shoulder joint injury and associated underlying diseases (diabetes, hypertension, hypothyroidism, inflammatory/rheumatoid arthritis, connective tissue diseases, long-term use of steroids, etc.) were also recorded. At the same time, similar methods were adopted for patients in the control group as those in the CaT group. The presence, location, and size of the RCTs in shoulder joint MRI were evaluated, and their combined underlying diseases were assessed from their medical records.

### Statistical analysis

SPSS software was used for all statistical analyses (Ver. 22.0; SPSS Inc., Chicago, Illinois). A comparison was made between the CaT group and the control group for age, gender, underlying diseases (diabetes, hypothyroidism, inflammatory/crystalline arthritis, connective tissue disease, hypertension), side of shoulder joint disease (left or right), the occurrence of RCTs (no, yes), the size of RCTs (complete or partial), and the incidence, position, and size of RCTs. A paired t-test was used for continuous variables, and a chi-square test (or appropriate Fisher’s exact test) was used for categorical variables. The incidence, location, and distribution of CaT were expressed with frequency and percentage, and the relationship between CaT and RCTs was evaluated with a Spearman’s Rho test. *P* < 0.05 was considered statistically significant.

## Results

The age, gender, and side of shoulder disease of the patients in the control group were matched with those of the patients in the CaT group. There was no significant difference in age (average age was 49.5 years), gender (58.3% female patients), and side of shoulder disease (61.1% right side) (*P* > 0.05) (Table 1). The incidence of diabetes and hypothyroidism was higher in the CaT group (31.5% vs. 25.9%; 7.4% vs. 2.8%) than in the control group, as there was a known correlation between the two diseases and CaT. However, this study showed no significant statistical significance between the two groups (*P* > 0.05) (Table 1). No statistically significant difference was found in the incidence of other complications, such as inflammatory/rheumatoid arthritis and connective tissue disease, between the two groups (*P* > 0.05) (Table 1).

**Table 1:** Comparison of demographics and incidence of rotator cuff tear between the two groups

	n (216)	CaT group (108)	Control group (108)	*P* value
Age	49.5±10.7	49.5±10.7	49.5±10.8	1.0*
Gender				
Male	90	45	45	1.0**
Female	126	63	63	
Complications				
Diabetes	62	34	28	0.367**
Hypothyroidism	11	8	3	0.122***
Inflammatory/Rheumatoid Arthritis	6	4	2	0.445***
Connective tissue disease	0	0	0	1.0***
Hypertension	27	15	14	0.842**
Nature of work				0.880**
Light physical labor	155	78	77	
Heavy physical labour	61	30	31	
Diseased side of shoulder joint n (%)				0.781**
Left	86	42	44	
Right	132	66	64	
Rotator cuff tear n (%)				0.023**
Yes	61	23	38	
No	155	85	70	
Size of rotator cuff tear (%)				0.422**
Partial tear	47	19	28	
Full-thickness tear	14	4	10	
Site of rotator cuff tear (%)				0.247***
Supraspinatus muscle	40	16	24	
Infraspinatus muscle	14	3	11	
Subscapular muscle	7	4	3	

*P *< 0.05

*Calculate the *P* value using the paired T-test

**Calculate the *P* value using the chi-square test

***Calculate the *P* values using Fisher’s exact test

The incidence of RCTs in the control group (37.2%) was significantly higher than that of the CaT group (23.4%) (*P* < 0.05) (Table 1), indicating that RCTs were more common in the control group. No statistically significant difference was observed in the size of RCTs between the two groups, but partial tears were more common for the two groups (*P* = 0.422) (Table 1). No statistically significant difference was found in the location of RCTs between the two groups (*P* = 0.569), and a significant proportion (65.6%) of RCTs were seen in the supraspinatus tendon. Meanwhile, in this study, it was found that tendon calcification was most commonly seen in the supraspinatus tendon (50.9%) and subscapular tendon (28.7%). The incidence of calcification in the subscapular tendon was relatively low (19.5%) and least on the teres minor tendon (0.9%) (Table 2). Among the 23 patients with CaT with RCTs in the CaT group, 14 cases (12.9%) had calcification locations on the tendon. Among the 10 patients with calcification and RCTs in the same tendon, only four (3.7%) had calcification and tears in the same location of the same tendon. There was no significant correlation between calcification site and RCTs in the CaT group (*P* > 0.05, r = 0.03) (Table 3).

**Table 2:** Distribution and percentage of rotator cuff tears in 108 patients in the CaT group

Site of rotator cuff tear	n	Percentage (%)
Supraspinatus muscle	55	50.9
Infraspinatus muscle	31	28.7
Subscapular muscle	21	19.4
Teres minor tendon	1	0.9
Total	108

**Table 3:** Correlation between calcification site and rotator cuff tears in the CaT group

Rotator cuff tear	n	Percentage (%)
No tear	84	77.8
Different tendons	14	12.9
Different positions on the same tendon	6	5.6
Same tendon in the same position	4	3.7
Total	108	

## Discussion

The correlation between CaT and RCTs has become a bone of contention in recent years. The correlation between the two can be predicted based on four aspects: CaT and RCTs have a similar pathogenesis, RCTs lead to CaT, CaT may cause cuff tears, or there is no correlation between RCTs and CaT [11].

The pathogenesis of CaT remains unknown. Initially, CaT was thought to be caused by the accumulation of hydroxyapatite in the rotator cuff tendon caused by degeneration and local necrosis of the tendon fibers and the increase of calcium phosphate content [13]. Recent studies have shown that the metaplasia mechanism of CaT is that tendon fibers transform into fibrocartilage and are calcified [14]. It has been reported in subsequent studies that metaplasia of tendon fibers, differentiation of tendon stem cells into chondrocytes and osteoblasts [15], endocrine disorders (thyroxine, estrogen, insulin) [16], and genetic factors [17, 18] may also be related to the development of CaT. In this study, the incidence of diabetes and hypothyroidism was high, but this high incidence was not statistically significant in the two groups of patients. Archer et al. found that calcified fibrocartilage in CaT does not have typical immunohistochemical markers formed by chondrocyte-mediated calcium and questioned the metaplasia mechanism of tendon fibers to transform into fibrocartilage and finally calcify [19].

**Fig. 2:**
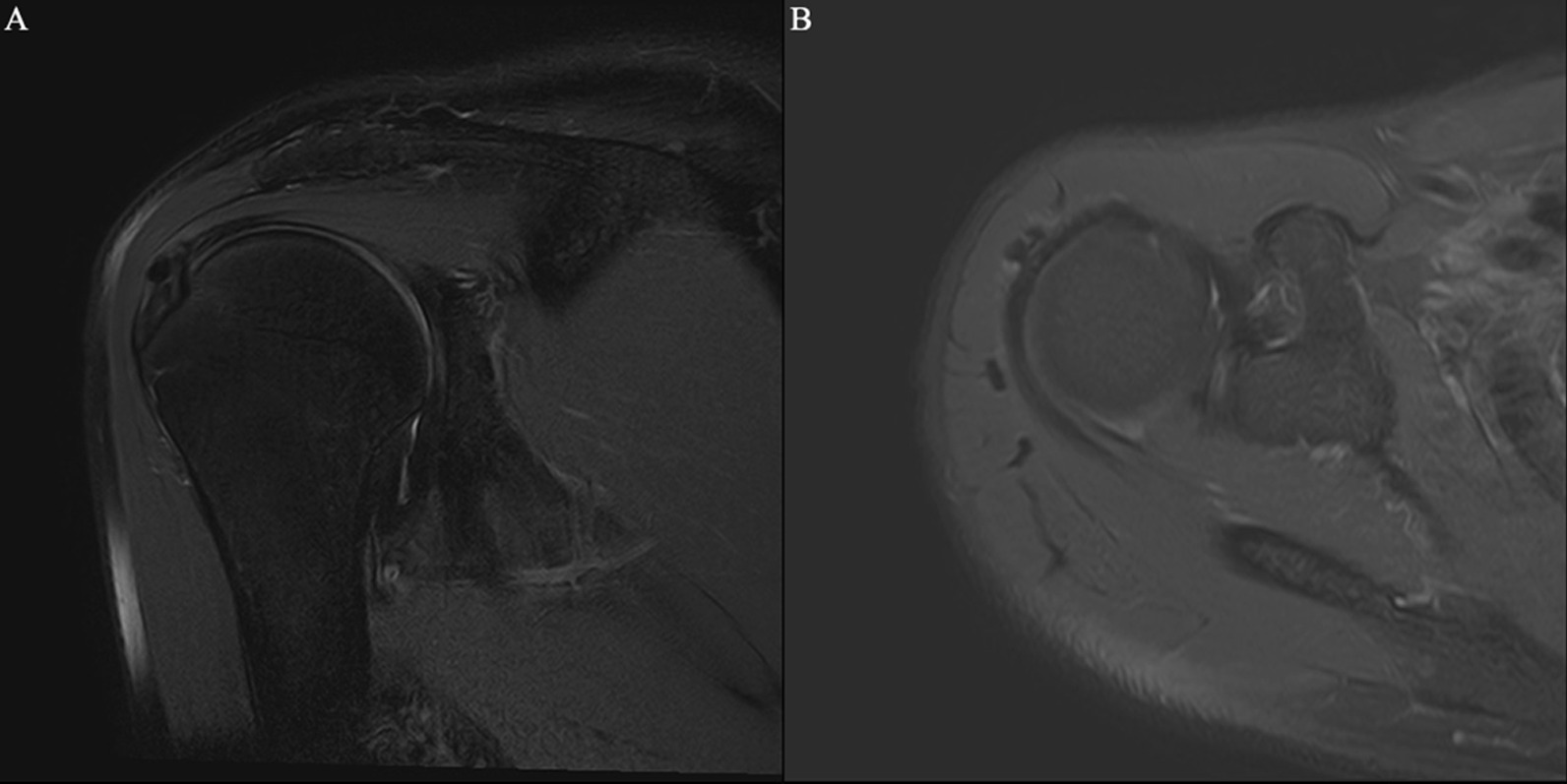
Focal tendinitis of the supraspinatus tendon combined with calcific tendinitis at attachment of nodule. *Note* Male, 46 years old, with periarthritis of shoulder. **A** T2-SPAIR oblique coronal position, **B** T2-SPAIR axial position: nodular low-signal calcification can be seen in tendons at attachment of nodule in the supraspinatus tendon, focal edema signals can be seen inside the tendon, and no tear was seen in the tendon

**Fig. 3:**
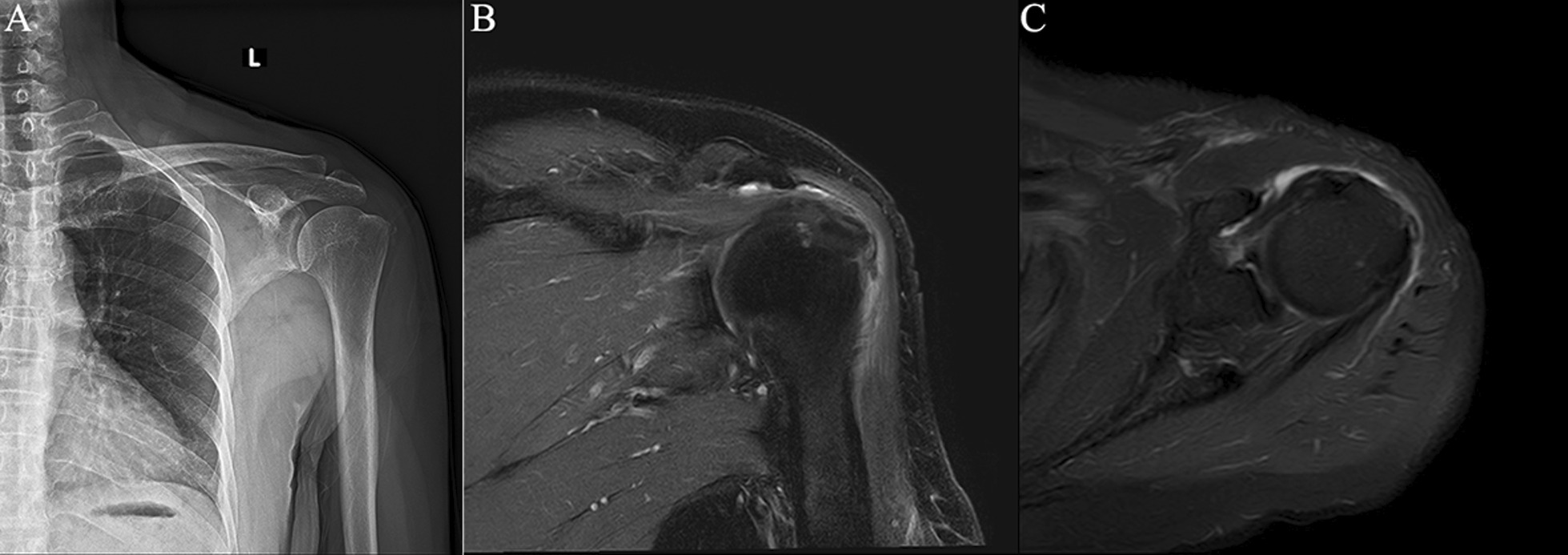
Diffuse tendinitis of the supraspinatus tendon combined with calcific tendinitis of the subspinatus at attachment. *Note* Female, 68 years old, with shoulder periarthritis. **A** calcifications can be found around the greater tuberosity of humerus on the X-ray film of the left shoulder joint; **B** T2-SPAIR oblique coronal position, **C** 2-SPAIR axial position: signs of diffuse mild edema can be observed in the supraspinatus tendon, combined with low signal calcification at attachment of the infraspinatus muscle of the greater tuberosity

**Fig. 4:**
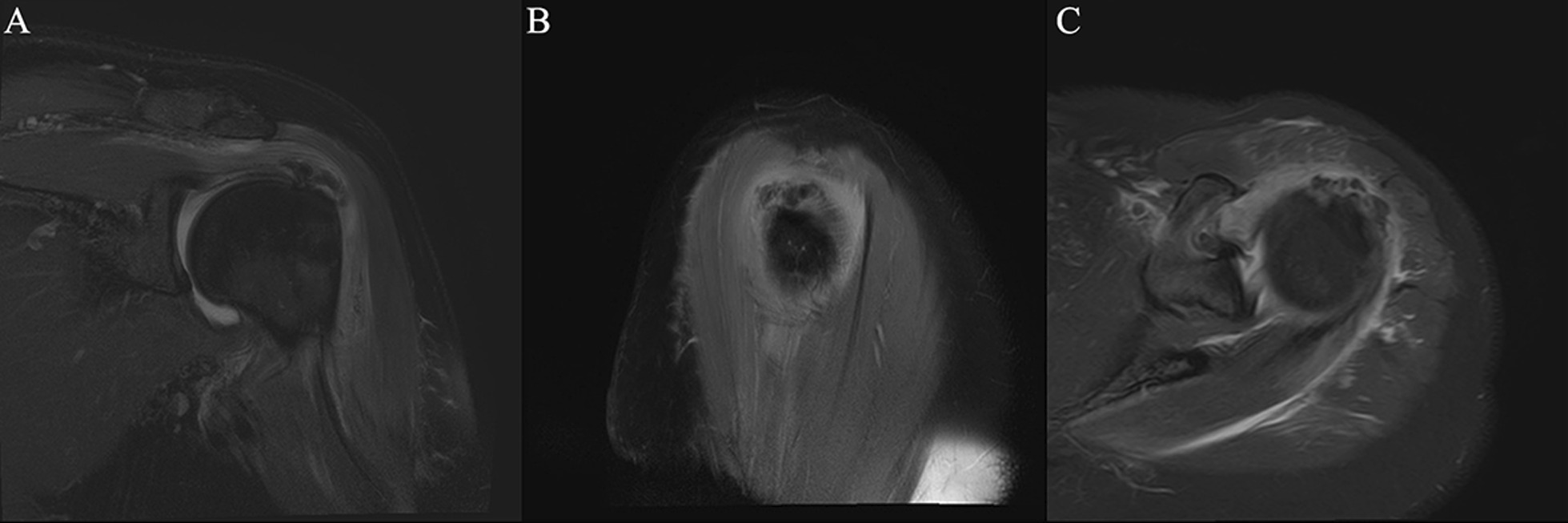
Calcific tendinitis of supraspinatus tendon combined with tear at attachment and tendon contracture at the broken end. *Note* Female, 43 years old, with shoulder periarthritis. **A** T2-SPAIR oblique coronal position, **B** PDW-SPAIR oblique sagittal position, **C** T2-SPAIR axial position: low-signal calcification can be seen in tendon at attachment of nodule in supraspinatus tendon and focal edema signals can be seen inside the tendon, irregular tear of tendon at attachment of nodule, and the tear range covers the tendon in the calcified area, accompanied by tendon contracture at the broken end

**Fig. 5:**
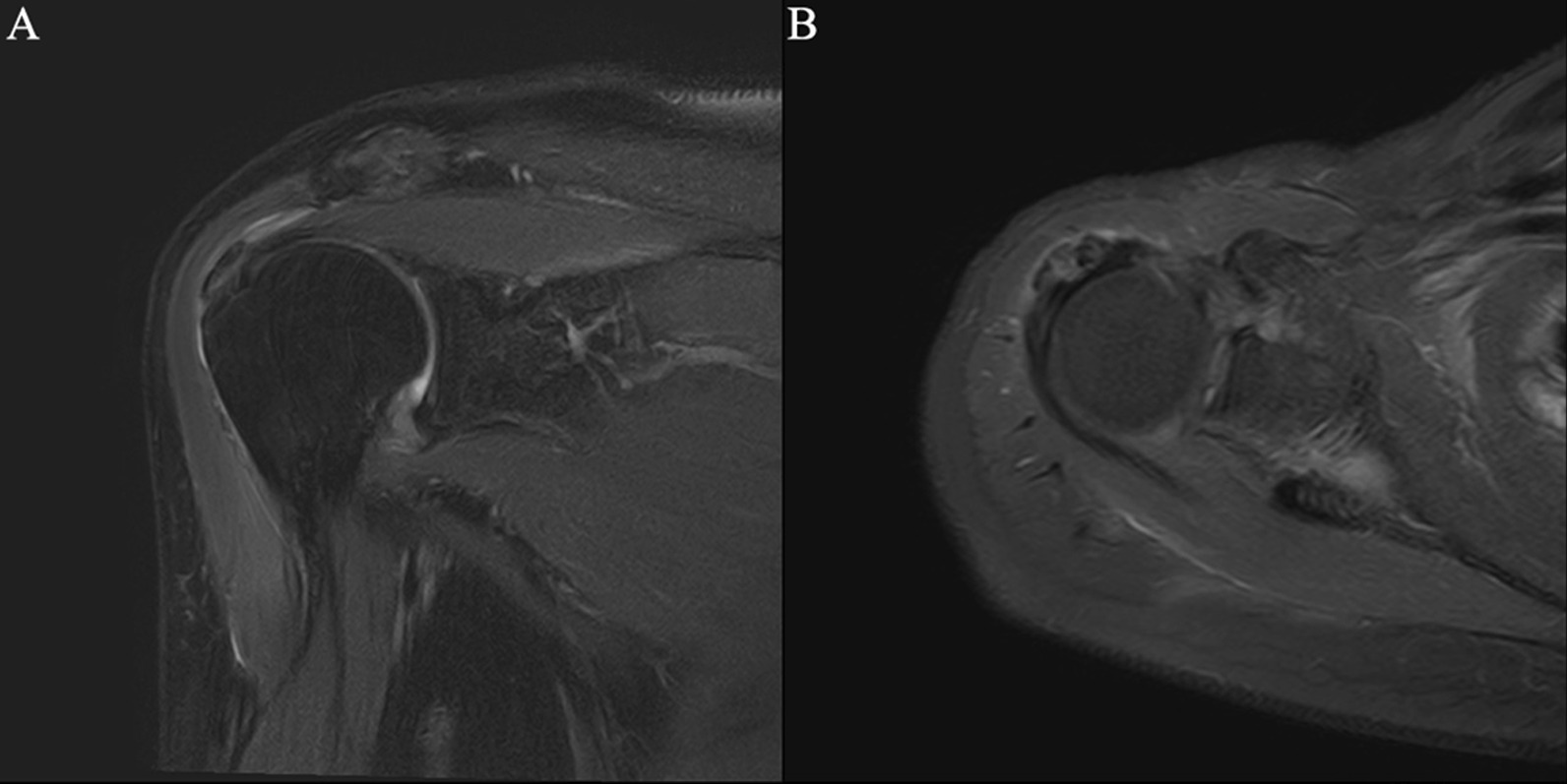
Calcific tendinitis of supraspinatus muscle with a partial tear of the capsular margin of the joint at attachment of nodule. *Note* Female, 55 years old, with rotator cuff injury. **A** T2-SPAIR oblique coronary position, **B** T2-SPAIR axis position: low-signal calcification can be seen in tendons at attachment of nodule in supraspinatus tendon and signs of focal edema can be seen inside the tendon, combined with partial tear of capsular tendon at attachment of nodule

**Fig. 6:**
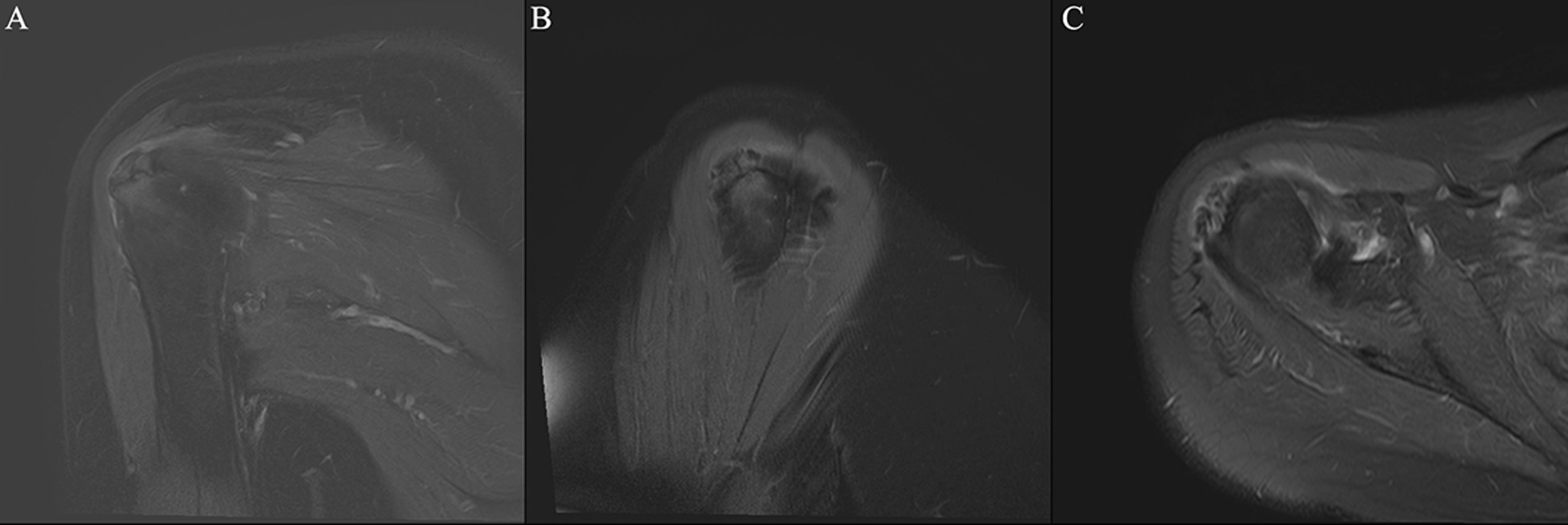
Diffuse tendinitis of the supraspinatus muscle combined with calcific tendinitis, partial tear of the tendon at attachment of nodule, combined with hyperostosis of greater tuberosity of humerus and focal bone marrow edema. *Note* Female, 55 years old, with shoulder arthritis. **A** T2-SPAIR oblique coronal position, **B** PDW-SPAIR oblique sagittal position, **C** T2-SPAIR axial position: low-signal calcification can be seen in tendons at attachment of nodule in supraspinatus tendon and signs of focal edema can be seen inside the tendon, partial tear of the tendon at the attachment of nodule, and the range of the tear covers the tendon in the calcified area, combined with hyperostosis of greater tuberosity of humerus and focal bone marrow edema

**Fig. 7:**
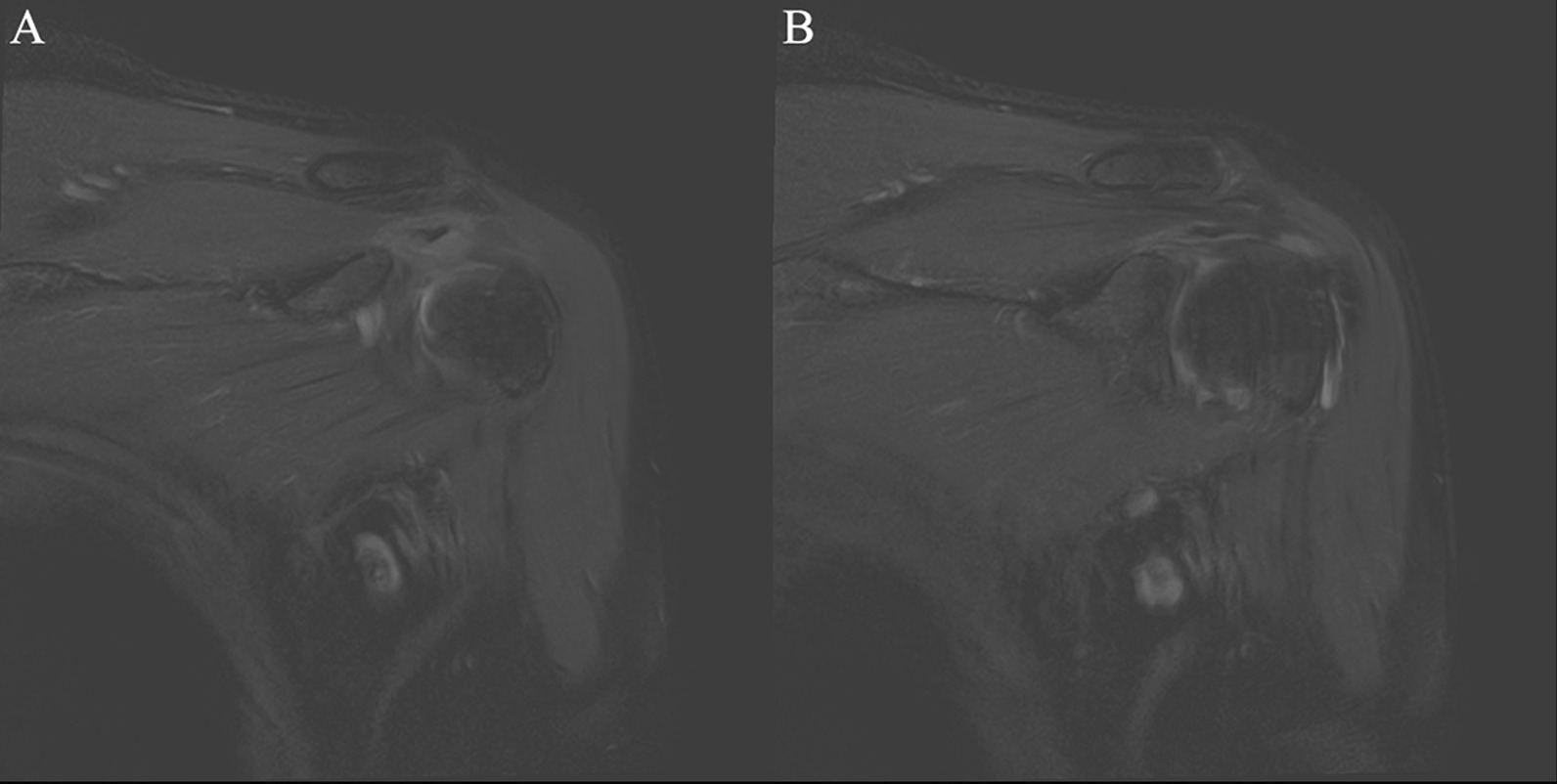
Tendinitis at tendon-muscle belly junction of supraspinatus muscle. *Note* Female, 52 years old, with shoulder periarthritis. **A**, **B** T2-SPAIR oblique coronal position: low-signal calcification can be seen at the junction tendon-muscle belly of of the supraspinatus muscle, and signs of edema can be seen inside the tendon

The pathogenesis of RCTs includes both external and internal factors. External factors include trauma, chronic impact, diabetes, and obesity, which can increase tendon damage. Internal factors include inadequate blood perfusion, tendon aging or degradation, apoptosis theory, and modification and calcification of the extracellular matrix [4, 20]. The most common cause of RCTs is tendon degeneration [11, 20]. Tendon degeneration is prevalent in patients with RCTs, and it is related to the reduction of tendon vessels [21]. In the rotator cuff, a relatively avascular region is called the avascular critical zone, a common site for the development of rotator cuff degeneration and tears [21, 22] and is also considered a common site for the development of rotator cuff calcification [21]. Therefore, some scholars believe that CaT is related to rotator cuff degenerative tears [5]. However, in this study, it was found that for the majority (95.7%) of patients with RCTs, the tears were found in different tendons or different areas of the same tendon (Figs. 2, 3, 4, 5, 6, 7).

Clinically, CaT mainly occurs in patients aged 30–60 years [23]. However, as patients age, the incidence of degenerative RCTs increases, too [24]. CaT can be observed, not only in the supraspinatus and subscapular tendon within the “avascular critical zone,” but also in the subscapularis and teres minor tendon outside the “avascular critical zone” [21]. Although CaT primarily occurs in the “avascular critical zone,” it is not uncommon that CaT affects other areas of the rotator cuff, or its calcification is limited to the deltoid muscle, but no tendon is damaged.

Our results also verified that 16% of patients with CaT had calcification in the subscapular tendon, and 0.7% of patients had CaT in the teres minor tendon. At the same time, calcification and tears were found to occur in different tendons or different areas of the same tendon. Calcification could also be seen in tendons outside the avascular critical zone, suggesting that the etiology of CaT was different from that of RCTs.

Although clinical evidence suggests that CaT associated with hydroxyapatite deposition is not a precursor or sequelae of degenerative tendon tears, there is still a significant correlation between rotator cuff tendon calcification and RCTs on histological and macroscopic levels [7]. However, the calcification deposited on the site of the RCTs appears to be histologically different from the calcification in hydroxyapatite deposition. Some scholars have found that 33% of patients with degenerative RCTs have obvious calcification at the site of tendon tears [25]. However, the calcifications associated with degenerative RCTs show different phosphorus concentrations and calcium: phosphorus ratios, suggesting that different calcium compounds may be responsible for the calcification of degenerative RCTs. In contrast, the phosphorus concentration and calcium: phosphorus ratios of all patients with CaT are consistent with hydroxyapatite crystals.

However, some studies suggest that CaT may accelerate the progress of RCTs. Merolla et al. [12] found that previous abnormal calcification may lead to or aggravate RCTs, which requires surgical treatment. Similarly, Ari et al. [5] reported that of 81 CaT patients who underwent arthrography, 27% had partial or complete RCTs. It is worth noting that in the study of Ari et al., the average age of patients was over 61 years old, which is the most common age for getting degenerative RCTs. This indicates that in their studies, the high prevalence of RCTs may be related to the age of the studied population, not just because of the complication of rotator cuff calcification. At the same time, some studies related to rotator cuff surgery have found that RCTs rarely occur even when patients only have CaT [9]. Other studies have shown that 23% of patients with RCTs will suffer CaT [7]. These conclusions are based on the results of research related to rotator cuff surgery, and only patients who fail to be cured with conservative treatment will undergo surgical intervention. Therefore, it is not entirely correct to conclude that CaT is correlated with RCTs. Therefore, for all patients with shoulder pain, MRI evaluation is more objective and accurate for assessing the correlation between CaT and RCTs [12]. The signal intensity of calcification on MRI is low, and the accuracy of identifying calcification with MRI is about 95% [12, 26]. In addition, the studies of Norenberg et al. [27] show that compared with X-ray, MRI boasts a diagnostic sensitivity of 98% and a specificity of 96% for shoulder joint calcification.

In the past, RCTs were rarely associated with CaT in imaging diagnosis. In a study using ultrasound to evaluate 94 rotator cuff CaT patients with an average age of 57 years, no RCTs occurred in the calcified region [10]. Beckmann et al. [11] conducted MRI examinations on 86 patients with CaT and 86 patients with shoulder joint pain in the control group, and the results showed no statistically significant difference in RCTs incidence between the two groups. In addition, in the CaT group, only 3/8 of the patients (3.5% of all CaTs) had calcifications and RCTs on the same tendon, which appeared in the same location of the tendon. In our study, only 4.3% of patients in the CaT group had calcifications and tears in the same area of the same tendon. Therefore, these research results support the view that CaT is not caused by RCTs, and it does not exacerbate RCTs. But there is possibility that CaT can cause tendon rupture indirectly by long-term mechanical change.

At present, this study still has some shortcomings. (1) The sample size is not big enough. For the correlation between CaT and RCTs to be accurately analyzed, the sample size needs to be further expanded. (2) In this study, MRI, rather than surgery, was adopted as the gold standard for diagnosing RCTs. As a result, some tiny tears in the tendon may be ignored. (3) This study evaluated the correlation between CaT and the current and static state of the tendon. It did not assess whether CaT increases the risk of tendinosis or tendon tears over a long period. (4) This study is based on using MRI to diagnose CaT, but calcification is not confirmed on x-ray. However, for all cases with x-rays, calcification can be seen through radiology.

## Conclusions

Compared with patients with shoulder pain suffering no CaT, patients with rotator cuff CaT are not subjected to increased risk of RCTs on MRI. Since different pathological processes may cause CaT and RCTs, there is no significant correlation between the two.

## Data Availability

Data related to the current study are available from the corresponding author on reasonable request.

## Abbreviations

RCTs: Rotator cuff tears
CaT: Calcific tendinitis
MRI: Magnetic resonance imaging
